# Arbuscular mycorrhizal fungi associated with maize plants during hydric deficit

**DOI:** 10.1038/s41598-023-28744-4

**Published:** 2023-01-27

**Authors:** Letícia Rezende Santana, Lais Noamy da Silva, Germanna Gouveia Tavares, Priscila Ferreira Batista, Juliana Silva Rodrigues Cabral, Edson Luiz Souchie

**Affiliations:** 1 Instituto Federal Goiano, Campus Rio Verde, Rodovia Sul Goiana km 01, Cx. P. 66., Rio Verde, GO CEP 75901-970 Brazil; 2 Faculdade de Agronomia, Universidade de Rio Verde, Fazenda Fontes do Saber - Campus Universitário, Cx Postal: 104, Rio Verde Goiás, CEP 75901-970 Brazil

**Keywords:** Climate change, Environmental biotechnology, Applied microbiology

## Abstract

The objective of this study was to verify the physiological behavior and development of maize plants under hydric deficit inoculated with the AMF *Rhizophagus clarus* and *Claroideoglomus etunicatum* and the commercial inoculant ROOTELLA BR in nonsterilized soil as a strategy to mitigate the effects of drought in the crop. Corn seeds were grown and inoculated with *R. clarus*, *C. etunicatum* and the commercial inoculant ROOTELLA BR separately at sowing. The plants were grown in a greenhouse and submitted to water deficit in stage V3, keeping the pots at 20% field capacity for 10 days. The first analyses were performed, followed by reirrigation for 2 days, and the analyses were performed again. The experiment was a double factorial, with 2 water treatments (irrigated and water deficit) × 4 inoculation treatments (control, ROOTELLA BR, *R. clarus, C. etunicatum*) × 5 replicates per treatment, totaling 40 vessels. The results indicate that the plants were able to recover favorably according to the physiological data presented. It is noted that in inoculated plants, there was no damage to the photosynthetic apparatus. These data demonstrate that AMF contribute greatly to better plant recovery after a dry period and a new irrigation period. Inoculation with AMF favors postwater stress recovery in plants.

## Introduction

Corn is an essential cereal in the global feeding of humans, in addition to being important for the production of ethanol, corn starch syrup and animal feed^1^. Because it is a C_4_ plant_,_ corn has greater efficiency in water use than C_3_ plants; because of their functional anatomy, they are considered more naturally evolved and able to concentrate more CO_2_ in cells, making photosynthesis a more effective process^2^. This is because Rubisco's CO_2_ concentration mechanism maintains a high CO_2_/O_2_ ratio and reduces photorespiration in plants with a type C_4_ photosynthetic apparatus. This high CO_2_/O_2_ ratio is due to the high affinity of PEPcase for CO_2,_ which in turn fixes carbon dioxide through the formation of oxalic acid and carries this four-carbon product to the cells of the vascular sheath, where it is decarboxylated. Rubisco is then used to repair CO_2_ and increase its concentration in the medium, causing Rubisco to operate at the limit of its maximum CO_2_ saturation rate_,_ inhibiting its oxygenation activity and eliminating photorespiration^3^.

Drought is one of the most serious environmental stresses that negatively affects plant growth and productivity. The reduced transpiration rate and active transport and membrane permeability changes result in limited absorption of water and nutrients by plants. This change affects nutrient metabolism, photosynthesis, respiration, growth regulation, and the decline in species growth^4^. Water deficit directly impairs the growth and development of plants^5^, and this damage causes the inhibition of photosynthesis, as there will be a reduction in the assimilation of CO_2_ due to stomatal closure, reducing the turgidity of guard cells, which is essential to keep the stomata open^6^.

There are soil microorganisms that are well known for having important effects on the ecosystem, such as arbuscular mycorrhizal fungi (AMF), which are essential partners for plants. Among many other benefits, they can alleviate the negative effects caused by water stress, aiding plant growth and development^7^. The tolerance to hydric stress of plants associated with AMF can be explained by the increase in root volume, with the development of external mycelium, resulting in a greater capacity to explore and absorb soil water by the hyphae, increase in leaf turgor, reduction of the osmotic potential, oxidative damage caused by reactive oxygen species (ROS), increased stomatal conductance and improved water use efficiency by plants and improved plant nutrition^8–11^.

In a study, mycorrhizal symbiosis increased plant biomass, chlorophyll concentration, and transpiration rate under dry conditions^12^. It has also been demonstrated that mycorrhizal association improves the hydraulic conductance of the root, gas exchange, osmotic adjustment^13^, and photosynthetic activities under hydric stress^14^. In addition, the tolerance to periods of increased water deficiency by AMF in host plants has other implicit mechanisms, such as water absorption through extraradical mycorrhizal hyphae, nutritional improvement, glomalin production in soil aggregate stability, antioxidant-protected systems, and aquaporin expression^15–21^.

Since maize is one of the most essential crops in the world and takes into account the increase in the world population, it is indispensable to improve the production and yield of the main crops under normal and dry conditions^7^. Thus, the objective of this study was to verify the physiological behavior and development of maize plants under hydric deficit inoculated with the AMF *R. clarus* and *C*.* etunicatum* and the commercial inoculant ROOTELLA BR in nonsterilized soil as a strategy to mitigate the effects of drought in the crop.

## Materials and methods

### Multiplication of non-commercial AMF

The AMF strains used in this study were multiplied by the availability of the inoculum *R*.* clarus* and *C. etunicatum* from the collection of the Soil Microbiology Laboratory of UNESP—Ilha Solteira, given to IF Goiano—Campus Rio Verde by the method of pot culture^22^.

The soil was collected in an area of IF Goiano Campus Rio Verde, used for multiplication, mixed with sand (3:1—soil:sand), and sterilized in an autoclave at 121 °C and 1.5 atm pressure. The process was repeated for 3 consecutive days and then dried in an oven at 100 °C for 24 h, and after being cold, it was moistened for the same period with distilled and sterilized water^23,24^.

*Urochloa ruziziensis* was used as a host plant, which was grown in a greenhouse under conditions irrigated with distilled water for 90 days and, after this period, subjected to hydric stress for 7 days to induce the proliferation of AMF spores^25,26^. Later, the plants were removed, a sample of the soil was collected, and the number of spores was evaluated according to^27,28^. The number of spores was determined on an acrylic plate with concentric rings under a SteREO Discovery. V8 stereomicroscope (Zeiss, Göttingen, Germany).

### Plant growth conditions and inoculation of AMF

The soil was collected in an area of IF Goiano—Campus Rio Verde. A soil sample was removed for chemical analysis (Supplementary Table S1) to observe the need for liming according to its base saturation. Subsequently, a mixture of soil (Red Latosol—typical soil of the Brazilian Cerrado savanna) and medium sand (3:1—soil:sand) and application of limestone (Filler dolomitic limestone 100% PRNT) was made in the soil with the aid of a concrete mixer, placed in 5-L pots, and taken to the greenhouse where the limestone reacted for 20 days until reaching saturation per base recommended for the crop (60%).

After 20 days, corn seeds (cv. Brevant B2360PW) were germinated in 5 L pots and grown in a greenhouse under natural conditions of light, relative humidity (65–85%), and an average temperature of 27 °C. Each pot was fertilized with 15 g of triple superphosphate, 2.5 g of potassium chloride, and 2 g of urea, based on the results of the chemical analysis (Supplementary Table S1) and the recommendations for Cerrado soils and corn^29^. Two weeks after planting, cover fertilization was performed with 1 g of urea and 1.5 g of potassium chloride per pot.

The mycorrhizal inoculant consisted of *R*.* clarus, C*.* etunicatum,* and the commercial inoculant ROOTELLA BR, which contains *Rhizophagus intraradices*, applied separately. The plants were inoculated in the sowing hole with 90 g of *R. clarus* (1.2 spores/g), 30 g of *C. etunicatum* (3.6 spores/g), and 0,0003 g of ROOTELLA BR (20.800 propagules/g with commercial measure of 120 g per hectare), separately.

### Induction of water stress in corn plants

The plants were distributed in the following treatments: (1) corn without inoculation (control) normaly irrigated, (2) corn without inoculation (control) with hydric deficit, (3) corn + *Rhizophagus clarus* normaly irrigated, (4) corn + *Rhizophagus clarus* with hydric deficit*,* (5) corn + *Claroideoglomus etunicatum* normaly irrigated, (6) corn + *Claroideoglomus etunicatum* with hydric deficit, (7) corn + ROOTELLA BR normaly irrigated, and (8) corn + ROOTELLA BR with hydric deficit. Each treatment consisted of five replicates containing one plant per pot. The control of the water content was performed through irrigation sensors, model 10 HS (METER Group, Inc. USA), once a day (in the morning) in the period before the imposition of the water deficit and twice a day in the deficit period (in the morning and late afternoon). The plants were submitted to water deficit in stage V3, when three leaves were fully developed, keeping the deficit vessels at 20% of the field capacity for 10 days, and the first analyses were performed. Then, the plants were reirrigated for 2 days at 80% of the field capacity, and analyses were performed again. Destructive analyses (water potential, mycorrhizal colonization, spore density, photosynthetic pigments, electrolyte leakage, and dry weight of leaves, roots, and total) were performed only after reirrigation.

### Measurement of water potential

The water potential (Ψw) was measured in the early morning in a Scholander pump after reirrigation. The determination consists of the collection of completely expanded leaves that were placed in the pressure pump chamber, where pressure was then applied until exudation occurred by cutting the petiole of the leaf for the reading of the applied pressure^30^.

### Physiological measurements

The analysis was performed using a system of determinations of infrared gas concentration (IRGA, Li-Cor—Li-6800). Parameters such as liquid photosynthetic rate *(A,* μmol CO_2_ m^−2^ s^−1^), stomatic conductance (*gs*, mol H_2_O m^−2^ s^−1^), internal concentration of CO_2_ (*Ci*, μmol CO_2_ mol^−1^), ambient concentration of CO_2_ (*C*a, μmol CO_2_ mol^−1^) and perspiration (*E,* mmol m^−2^ s^−1^) were determined in all treatments. An irradiance of 1000 μmol m^−2^ s^−1^ was used throughout the experiment. The measurements were performed from 8:00 to 11:00.

### Chlorophyll fluorescence measurements

Chlorophyll fluorescence measurements were performed using Irga, Li-Cor—Li-6800 equipment at 4:00 a.m. after leaf darkening for 40 min at room temperature to measure the minimum fluorescence (Fo) and maximum fluorescence (Fm). Thus, other parameters were obtained, such as the maximum quantum yield of PSII, calculated by Fv/Fm (Fv = Fm − Fo), while the actual quantum yield of PSII was calculated by ΦPSII = (Fm′ − F)/Fm′. In a state of adaptation to light, nonphotochemical quenching (qN) was calculated by qN = 1 − (Fm′ − Fo)/(Fm − Fo).

### Morphological characteristics

The height and diameter of the plants were obtained, and subsequently, the stems, leaves, and roots were separated and dried in an oven at 65 °C with forced air circulation to obtain the dry weight of leaves, roots, and total dry weight.

### Determination of chlorophyll concentration

The concentrations of carotenoids, chlorophyll *a,* and *b* were determined spectrophotometrically at 480 nm, 649.1 nm, and 665.1 nm, respectively, after pigment extraction from the leaf disc (~ 5 cm^2^) immersed in 5 mL of dimethyl sulfoxide (DMSO) with calcium carbonate (CaCO_3_) (50 g L^−1^) at 65 °C in a water bath. The disks remained in the solution for 24 h. The values were transformed to carotenoids, chlorophyll *a, b,* and totals in the leaves, expressed as μg cm^−2^^31^. Total chlorophyll was obtained by summing Chl*a* and Chl*b*.

### Observation of the association between fungi and corn roots

Root samples (~ 0.5 g) previously kept in 50% alcohol were depigmented by the modified method of^32^, in which the roots were immersed in KOH (10%) in a water bath at 90 °C for 60 min. Then, the roots were washed with distilled water and transferred to HCl solution (1%) for 5 min. Then, the HCl was removed, and the blue dye trypan (0.05%) was added to lactoglycerol and incubated for 10 min in a water bath at 90°C^33^ for root staining. To evaluate the mycorrhizal colonization rate, slides were made with root fragments, allowing the structures to be visualized under a Leica DM500 microscope, with a Leica ICC 50 camera adapted to the LAZ EZ software, version 1.8.0, following the quadrant intersection technique^34^.

### Spore density

The spore density in the soil of each vessel was determined using the wet sieving technique^27^. Thus, 100 g of soil was washed and sifted 6 times, placed in a Falcon tube with water, and centrifuged at 3000 rpm for 3 min. Then, water was added, and a 50% sucrose solution was added and centrifuged for another 2 min. Then, the liquid containing the spores was poured into the sieve to wash this sample and finally stored in a container until analysis in the laboratory. The number of spores was determined on an acrylic plate with concentric rings under a SteREO Discovery. V8 stereomicroscope (Zeiss, Göttingen, Germany).

### Electrolytic leakage

Five leaf discs were collected from completely expanded upper leaves and washed in deionized water. Then, electrolyte leakage was measured by measuring the free electrical conductivity of the bottle solution using a portable electrical conductivity meter, model TDS-3. After this first measurement, the vials were placed in a greenhouse at 100 °C for 1 h, and later, the second reading was made regarding total conductivity, adapted from^35,36^.

### Statistical analysis

The experiment was double factorial, with 2 water treatments (irrigated and water deficit) × 4 inoculation treatments (control, Rotella BR®, *Rhizophagus clarus, Claroideoglomus etunicatum*) × 5 replicates per treatment, totaling 40 vessels. The homogeneity of variance was confirmed by Bartlett’s test. The data were submitted to variance analysis, and the means were compared by the Tukey test (5%). The statistical procedures were performed using the Sisvar computer program 11^37^. Principal component analyses (PCAs) were run on the whole dataset. These analyses were run in the FactoMineR^38^ and factoextra^39^ packages in R software^40^. The data were first scaled using the scale function and then analysed using the PCA function.

### Ethics approval and consent to participate

We all declare that manuscript reporting studies do not involve any human participants, human data, or human tissue. Therefore, it is not applicable.

### Complies with international, national, and/or institutional guidelines

Experimental research and field studies on plants (either cultivated or wild) comply with relevant institutional, national, and international guidelines and legislation.

## Results

**Figure 1:**
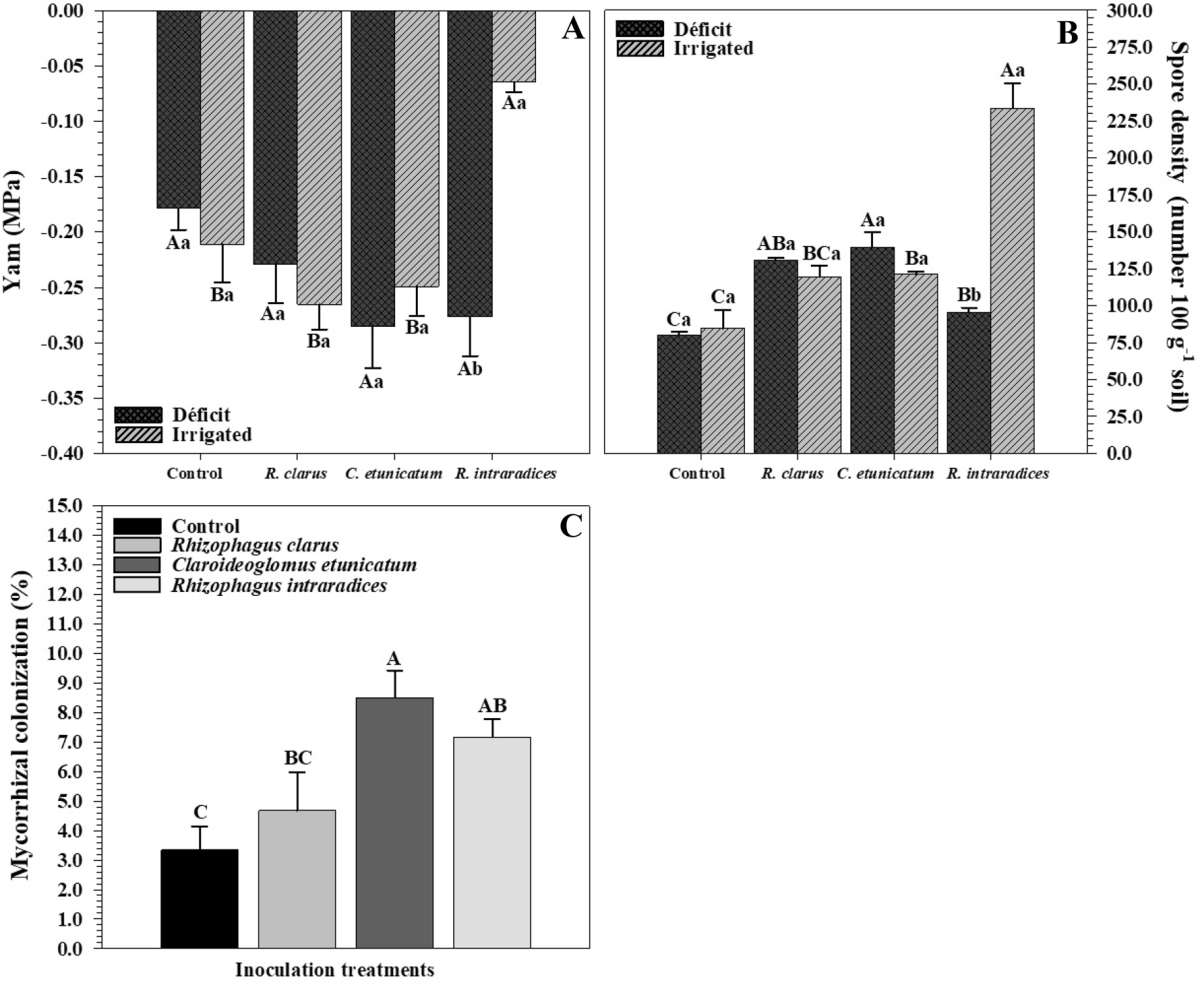
Water potential (**A**), spore density (**B**), and mycorrhizal colonization (**C**) of corn plants *(Zea mays)* submitted to different water treatments and inoculation with AMF after reirrigation. The bars represent the mean ± standard error of the mean (SEM) (*n* = 4). Means followed by the same letter, uppercase among inoculation treatments and lowercase between hydric treatments, do not differ (*p* > 0.05) among themselves by Tukey’s test.

The water potential (Ψw) measured after reirrigation and spore density had a significant interaction among the hydric and inoculation treatments (Fig. 1A,B). Plants under hydric deficit (HD) showed no difference. Plants irrigated and inoculated with *R. intraradices* (ROOTELLA BR) exhibited the highest water potential value (Ψw − 0.06), differing from the others. Analysing the hydric treatments within the inoculation levels, only the treatment inoculated with *R. intraradices* (ROOTELLA BR) differed between HD and irrigated plants, in which irrigated plants had higher Ψw than HD plants (Fig. 1A). The highest number of spores in HD treatment plants was observed in plants inoculated with *C. etunicatum* (139.66 100 g^−1^ soil), but this treatment did not differ from those inoculated with *R. clarus* (131 spores 100 g^−1^ soil). The lowest number of spores was present in plants without inoculation (79.66 100 g^−1^ soil) but did not differ from those inoculated with *R. intraradices* (ROOTELLA BR). Comparing the hydric treatments within each inoculation level, only those inoculated with *R. intraradices* (ROOTELLA BR) showed a difference in spore density, in which irrigated treatment plants were larger than water deficit plants (Fig. 1B).

Plants inoculated with *C. etunicatium* showed a higher mean colonization value (8.5%), followed by those inoculated with *R. intraradices* (ROOTELLA BR) (7.16%) and *R. clarus* (4.66%). Plants without inoculation had the lowest mycorrhizal colonization value (3.33%).

**Figure 2:**
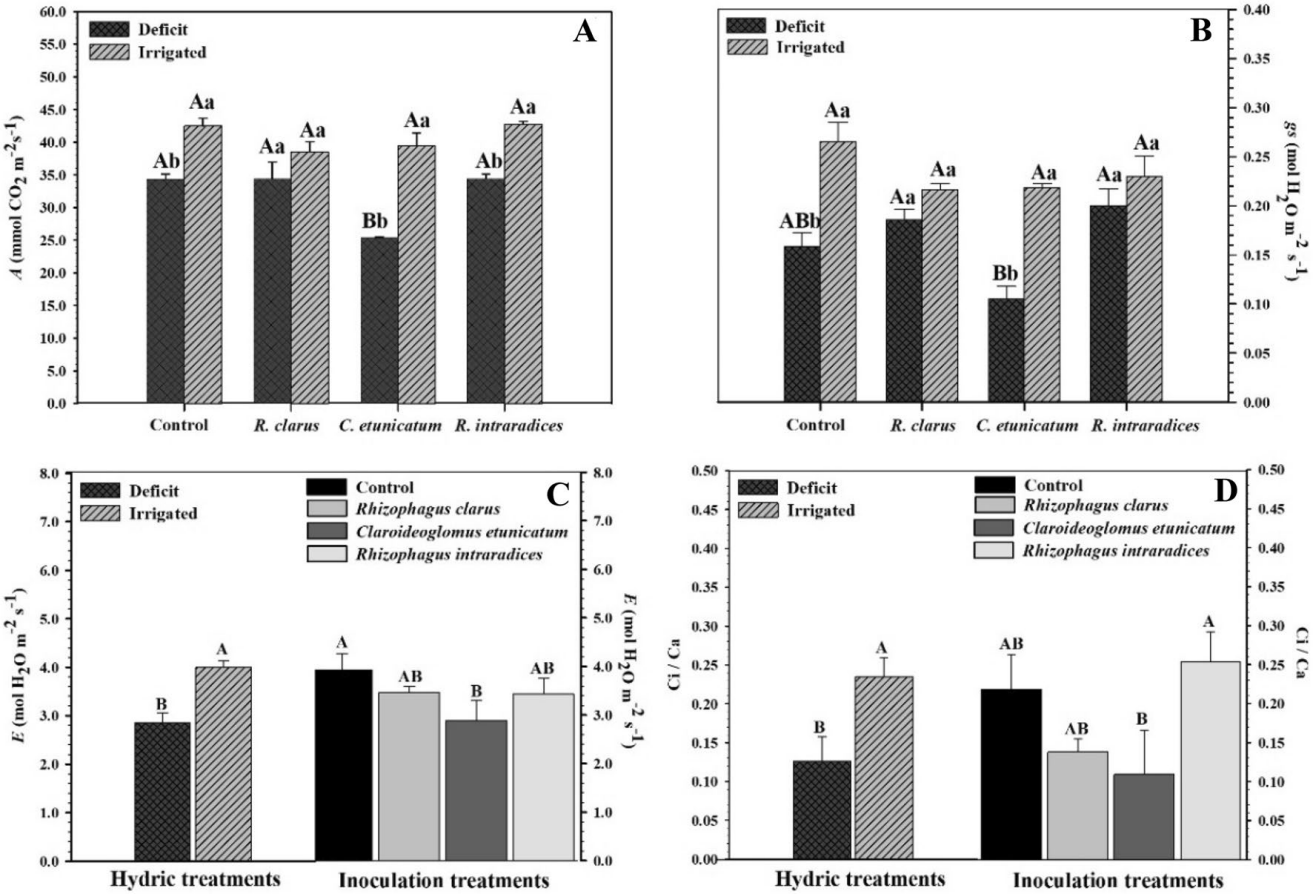
Photosynthetic rate (**A**), stomatal conductance (**B**), transpiration (**C**), and internal and external concentrations of CO_2_ (**D**) of corn plants *(Zea mays)* submitted to different water treatments and inoculated with AMF after reirrigation. The bars represent the mean ± standard error of the mean (SEM) (*n* = 4). Means followed by the same letter, uppercase among inoculation treatments and lowercase between water treatments, do not differ (*p* > 0.05) among themselves by Tukey’s test.

After reirrigation, the photosynthetic rate and stomatal conductance *(gs)* (Fig. 2A, B) also showed significant interactions among the hydric and inoculation treatments. Plants without inoculation and inoculated with *R. clarus* and *R. intraradices* (ROOTELLA BR) that experienced the deficit had the highest mean values of *A* (34.31 μmol CO_2_ m^−2^ s^−1^, 34.38 μmol CO_2_ m^−2^ s^−1^, and 34.43 μmol CO_2_ m^−2^ s^−1^, respectively), while plants inoculated with *C. etunicatum* had the lowest mean photosynthesis value (25.33 μmol CO_2_ m^−2^ s^−1)^. The photosynthetic rate of irrigated plants showed no difference between inoculation treatments. Analysing the hydric treatments within the inoculation levels, plants without inoculation showed a higher mean value in the irrigated treatment (42.48 μmol CO_2_ m^−2^ s^−1^). The treatment inoculated with *R. clarus* showed no difference between the deficit (34.38 μmol CO_2_ m^−2^ s^−1^) and irrigated (38.5 μmol CO2 m^−2^ s^−1^) treatments. For plants inoculated with *C. etunicatum* and *R. intraradices* (ROOTELLA BR), the highest values were observed for irrigated treatment plants (Fig. 2A).

The stomatic conductance *(gs)* in the HD treatment had the highest values in inoculated plants with *R. clarus* (0.18 mol H_2_O m^−2^ s^−1^) and *R. intraradices* (ROOTELLA BR) (0.20 mol H_2_O m^−2^ s^−1^) but did not differ from those without inoculation (0.15 mol H_2_O m^−2^ s^−1^). The lowest value was for those inoculated with *C. etunicatum* (0.10 mol H_2_O m^−2^ s^−1^). Between hydric treatments within each inoculation, control plants and plants inoculated with irrigated *C. etunicatum* showed higher *gs* than HD plants. Inoculation with *R. clarus* and *R. intraradices* (ROOTELLA BR) showed no difference between water treatments.

Transpiration *(E)* showed differences in treatments separately (Fig. 2C). For hydric treatments, *E* was higher in irrigated plants (4.99 mol H_2_O m^−2^ s^−1^) than in plants under hydric deficit (2.85 mol H_2_O m^−2^ s^−1^). In the inoculation treatments, the highest mean value of *E* was for noninoculated plants (3.93 mol H_2_O m^−2^ s^−1^), which did not differ from those inoculated with *R. clarus* (3.46 mol H_2_O m^−2^ s^−1^) and *R. intraradices* (ROOTELLA BR) (3.43 mol H_2_O m^2^ s^−1^). The lowest value was for plants inoculated with *C. etunicatum* (2.88 mol H_2_O m^−2^ s^−1^). The efficiency of water use showed no difference between treatments.

The internal and external concentrations of CO_2_ (Ci/Ca) also differed separately for the treatments (Fig. 2D). In the hydric treatments, the highest mean value was observed in irrigated plants. In the inoculation treatments, the highest mean *Ci/Ca* occurred in plants inoculated with *R. intraradices* (ROOTELLA BR), which did not differ from those inoculated with *R. clarus* and from those not inoculated. The lowest value was for the inoculated with *C. etunicatum.*

**Figure 3:**
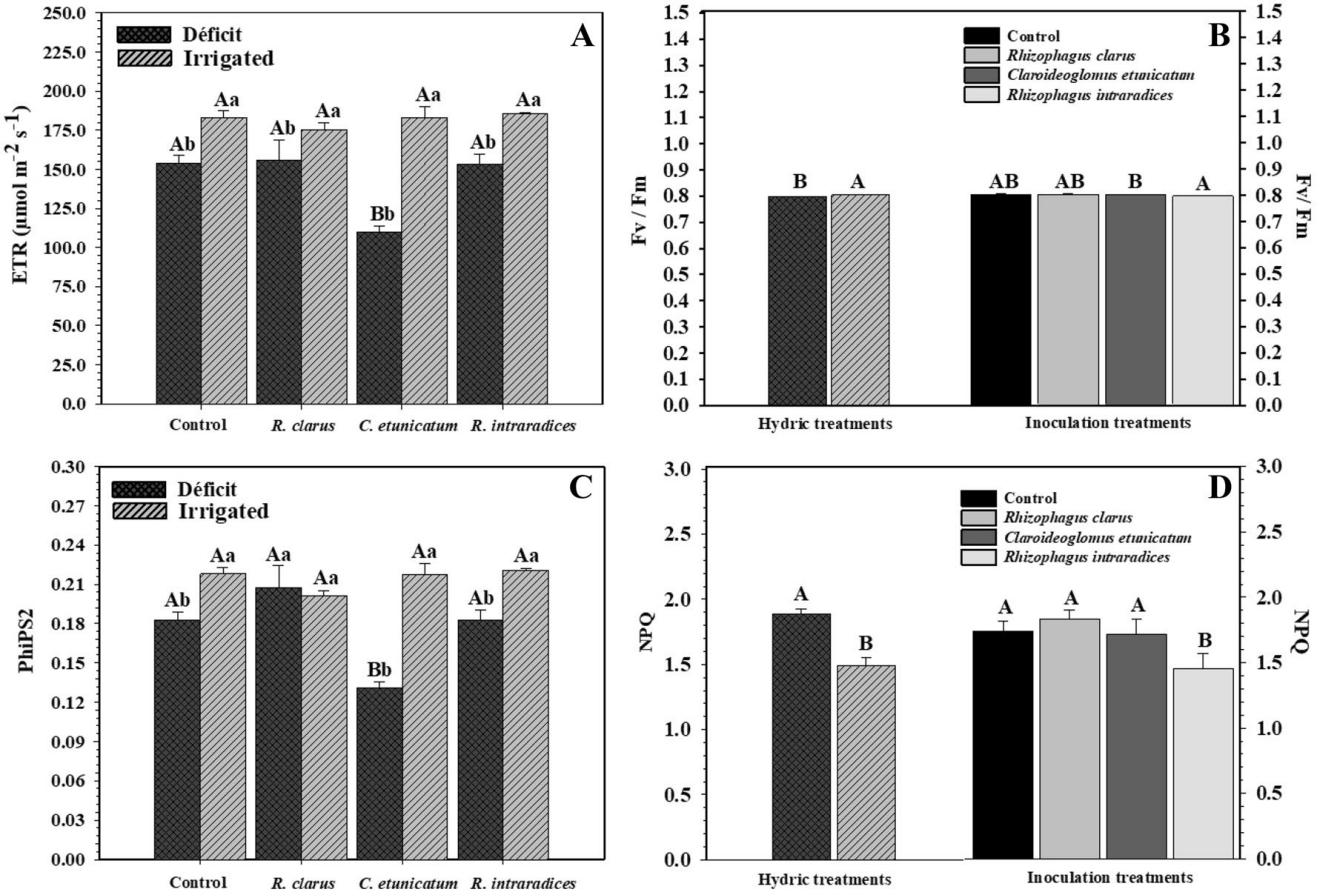
Electron transport [ETR] (**A**), maximum quantum yield of FS II [Fv/Fm] (**B**), effective photochemical efficiency of photosystem II [PhiPS2] (**C**), and nonphotochemical extinction coefficient [NPQ] (**D**) of corn plant*s (Zea mays)* subjected to different water treatments and inoculation with arbuscular mycorrhizal fungi in the period of water restriction. The bars represent the mean ± standard error of the mean (SEM) (*n* = 4). Means followed by the same letter, uppercase among inoculation treatments and lowercase between water treatments, do not differ (*p* > 0.05) among themselves by Tukey’s test.

Electron transport (ETR) differed among treatments (Fig. 3A). For HD plants, the lowest value was observed for plants inoculated with *C. etunicatum*. The other parameters did not differ. In the irrigated water treatment, the plants showed no difference. Observing the hydric treatments within the inoculation levels, all irrigated plants were larger than plants submitted to the hydric deficit. The maximum quantum yield of photosystem II (Fv/Fm) differed for the hydric treatments (Fig. 3B). HD plants were smaller than irrigated plants. Regarding inoculation treatments, plants inoculated with *R. intraradic*es had a higher mean value of Fv/Fm but did not differ from the other treatments.

Regarding PhiPS2, there was a difference between the hydric and inoculation treatments (Fig. 3C); HD plants without inoculation (0.18) and those inoculated with the AMF *R. clarus* (0.20) and *R. intraradices* (ROOTELLA BR) (0.18) showed the highest mean values, while plants inoculated with *C. etunicatum* exhibited the lowest value (0.13). In the irrigated treatment, there was no difference between the plants. Observing the hydric treatments in the different inoculations, noninoculated plants had a higher response in the irrigated treatment (0.21 for irrigated and 0.18 for HD), as well as those inoculated *with C. etunicatum* (0.21 for irrigated and 0.13 for HD). Those inoculated with *R. clarus* and *R. intraradices* (ROOTELLA BR) did not differentiate between hydric treatments (0.20 for both hydric treatments).

The nonphotochemical extinction coefficient (NPQ) differed separately for the hydric and inoculation treatments (Fig. 3D). Concerning hydric treatments, plants under HD presented a higher mean NPQ value. For inoculation treatments, control plants inoculated with *R. clarus* and *C. etunicatum* showed higher mean NPQ values. The lowest value was demonstrated by plants inoculated with *R. intraradices* (ROOTELLA BR).

**Figure 4:**
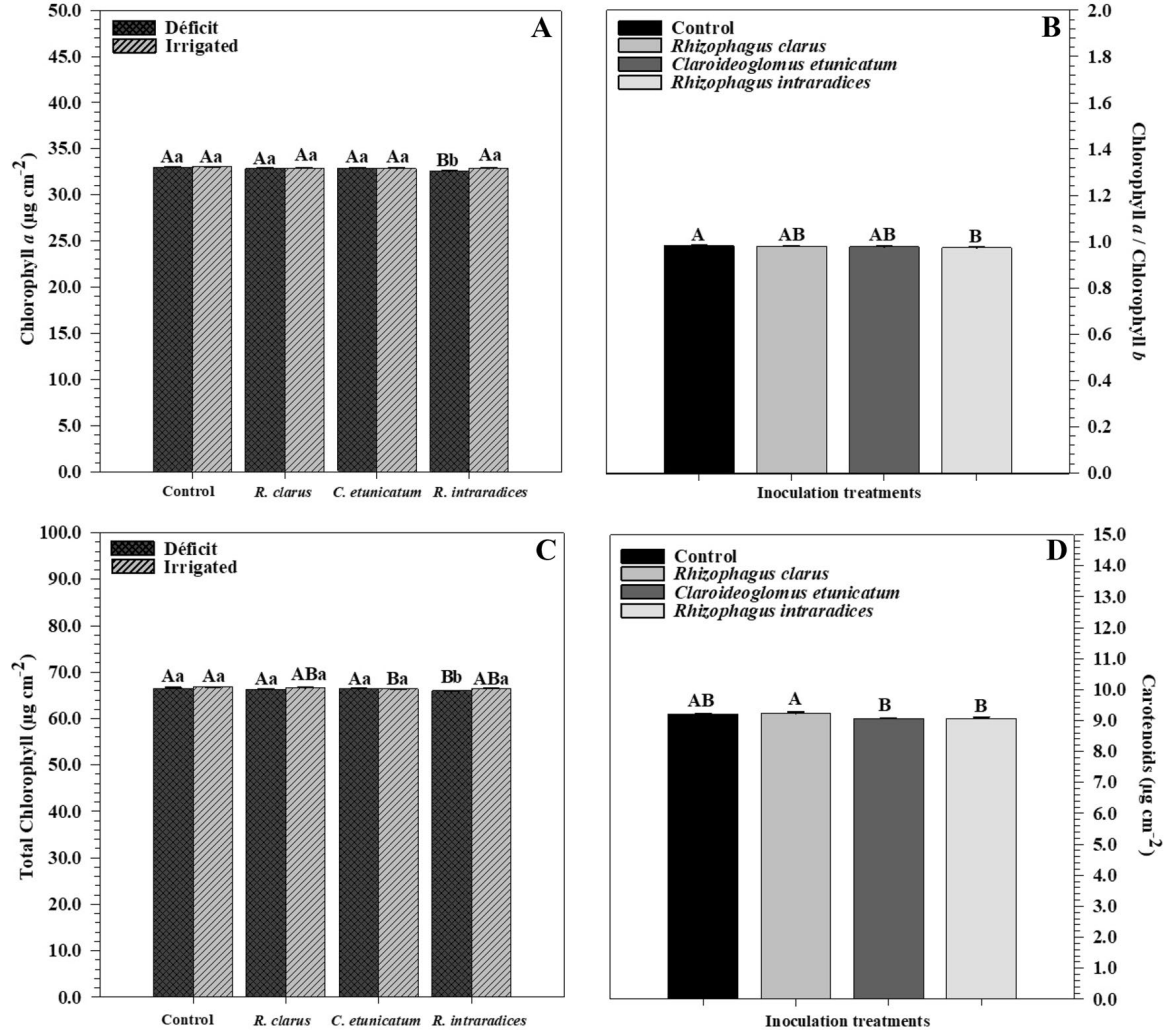
Chlorophyll *a* (**A**), Cl*a*/Cl*b* (**B**), total chlorophyll (**C**), and carotenoid (**D**) concentrations of corn plants *(Zea mays)* submitted to different water treatments and inoculated with AMF after reirrigation. The bars represent the mean ± standard error of the mean (SEM) (*n* = 4). Means followed by the same letter, uppercase among inoculation treatments and lowercase between water treatments, do not differ (*p* > 0.05) among themselves by Tukey’s test.

Chlorophyll concentration (Fig. 4A) differed among the inoculation and hydric treatment factors. Under hydric deficit, the highest pigment concentration was observed in control plants (32.94 μg cm^−2^) inoculated with *R. clarus* (32.85 μg cm^−2^) and *C. etunicatum* (32.88 μg cm^−2^), while plants inoculated with *R. intraradices* (ROOTELLA BR) had the lowest mean value (32.55 μg cm^−2^). In the irrigated treatment, the plants showed no difference. Analysing the hydric treatments within each inoculation level, only the treatment inoculated with *R. intraradices* (ROOTELLA BR) showed a difference, in which the hydric deficit plants had a lower value than irrigated plants. The chlorophyll *b* concentration showed no difference between the inoculation and water treatments. In the Cla/Clb ratio (Fig. 4B), the lowest mean value was for the inoculated with *R. intraradices* (ROOTELLA BR) but did not differ from the other treatments.

The total chlorophyll concentration (Clt) differed among treatments (Fig. 4C). For the WD treatment, only those inoculated with *R. intraradices* (ROOTELLA BR) differed, presenting a lower mean value of Clt. In the irrigated treatment, the highest mean value was for noninoculated plants but did not differ from those inoculated with *R. clarus* and *R. intraradices* (ROOTELLA BR), while the lowest value was for those inoculated with *C. etunicatum*. Observing the water treatments within the different inoculations, only those not inoculated and inoculated with *R. intraradices* (ROOTELLA BR) differed, in which the irrigated treatments were higher than those of HD. In the carotenoid (Fig. 4D), the lowest concentration was observed in those inoculated with *C. etunicatum* and *R. intraradices* (ROOTELLA BR), with an average value of 9.06 μg cm^−2^ for both, but did not differ from the other treatments.

**Figure 5:**
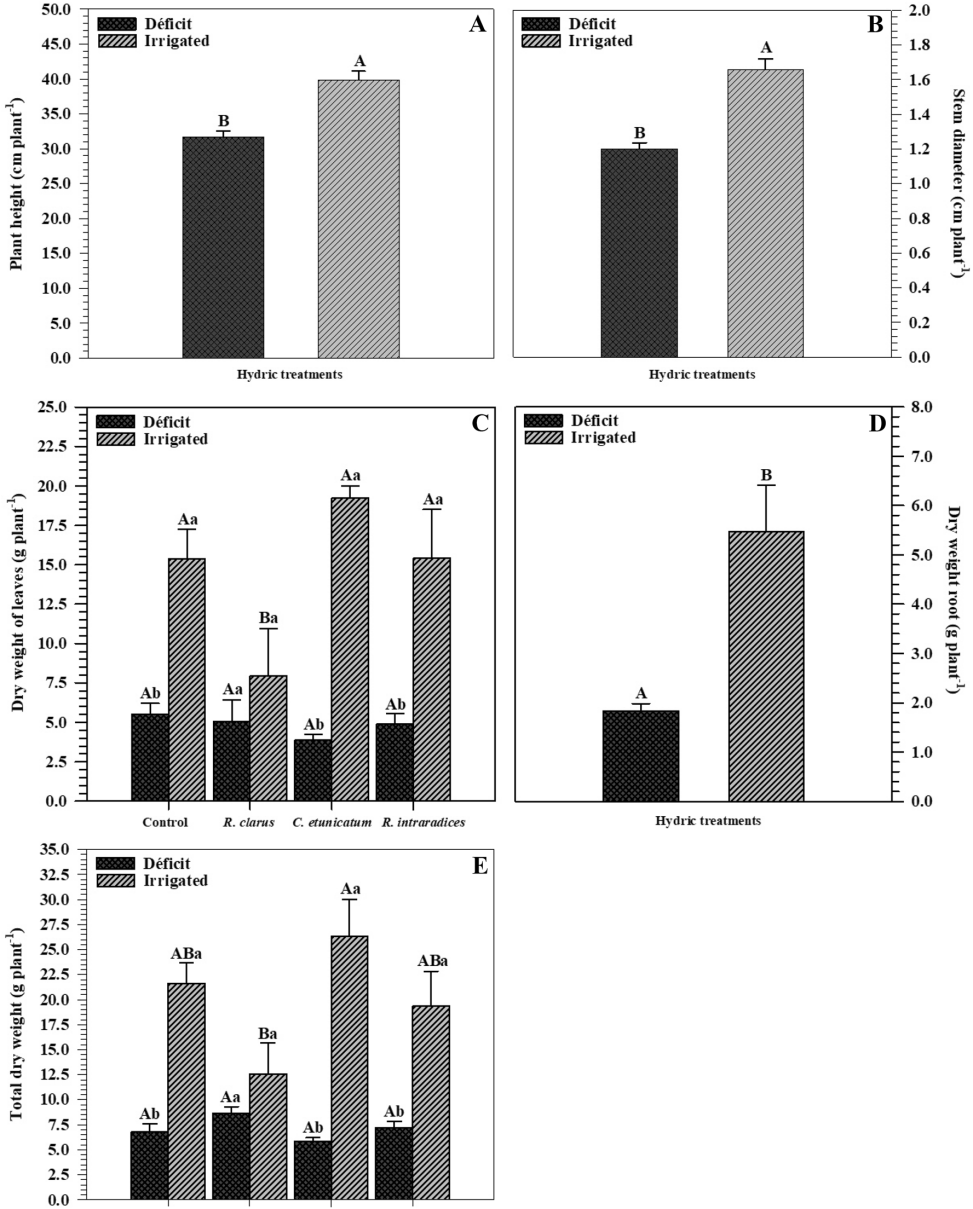
Plant height (**A**), stem diameter (**B**), dry leaf weight (**C**), dry root weight (**D**), and total dry weight (**E**) of corn plants *(Zea mays)* submitted to different water treatments after reirrigation. The bars represent the mean ± standard error of the mean (SEM) (*n* = 4). Means followed by the same letter, uppercase among inoculation treatments and lowercase between water treatments, do not differ (*p* > 0.05) among themselves by Tukey’s test.

The height (Fig. 5A) and stem diameter (Fig. 5B) of the plants showed differences separately only for the hydric treatments. The height of HD plants was lower (31.66 cm) than that of plants kept under irrigation (39.83 cm) (Fig. 5A). The stem diameter also presented the same response, in which HD plants had an average of 1.20 cm and irrigated plants reached an average of 1.65 cm.

The dry weight of the leaves evaluated after reirrigation differed among treatments (Fig. 5C), while HD plants did not differ from each other. For the irrigated treatment, plants inoculated with *R. clarus* had a lower average dry weight value. Analysing the water treatments within each inoculation, it was observed that all irrigated plants were larger than those of HD. The dry weight of the roots differed only in the hydric treatments (Fig. 5D), in which the dry weight of the irrigated plants was higher than that of the HD plants. The total dry weight differed among treatments (Fig. 5E). HD plants did not differ from each other. In the irrigated treatment, those inoculated with *C. etunicatum* had the highest mean value of total dry weight but did not differ from those inoculated with *R. intraradices* (ROOTELLA BR) and those not inoculated. The lowest total dry weight value was for those inoculated with *R. clarus.* Regarding the hydric treatments within the different inoculations, all irrigated treatments were higher than HD plants.

**Figure 6:**
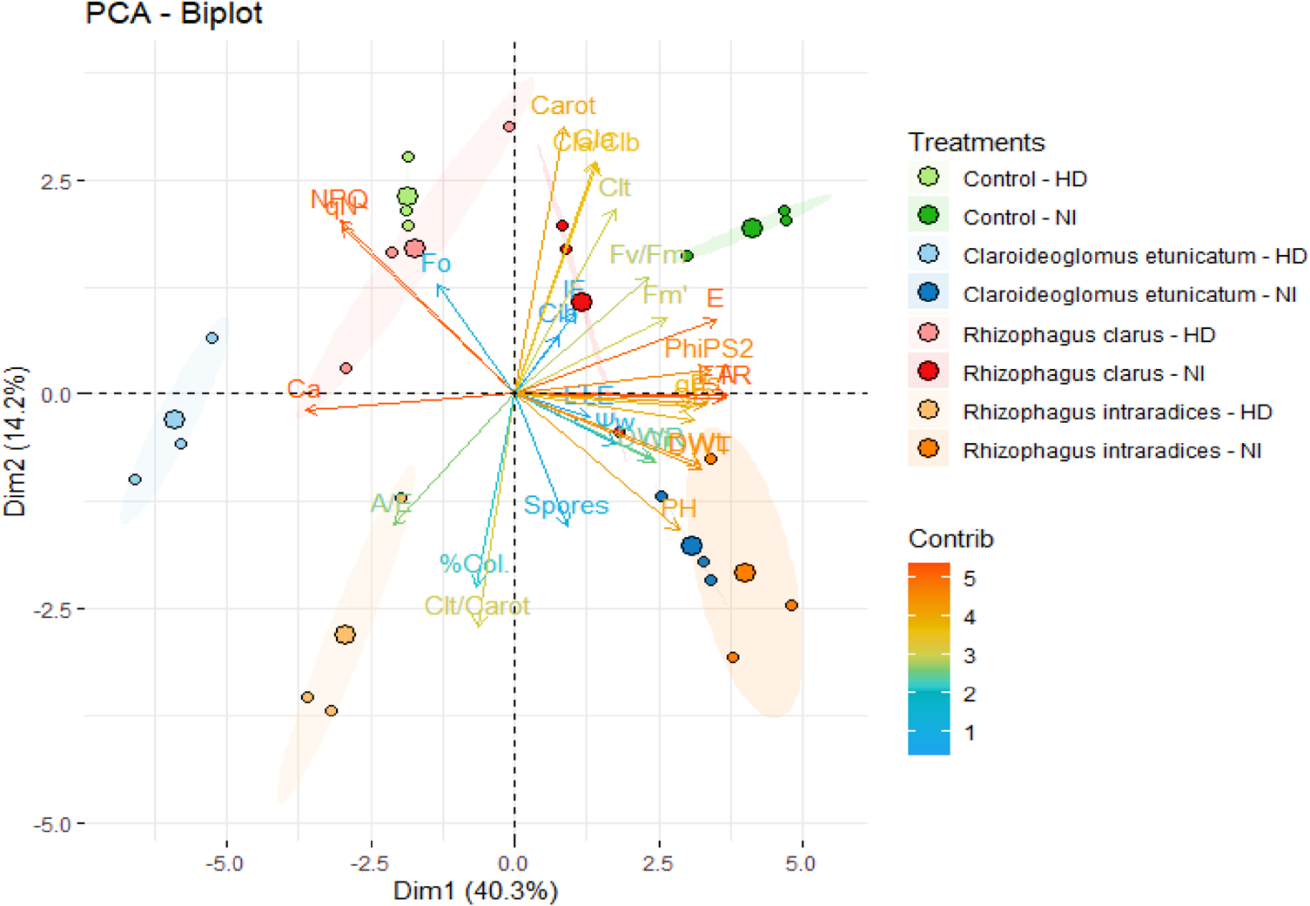
PCA biplot of the first and second dimensions of corn plants *(Zea mays)* submitted to different water treatments and inoculated with AMF after reirrigation. *HD* Hydric deficit and *NI* normaly irrigated.

The first two components explained 54,5% (PC1 40,3% and PC2 14,2%) of the variance, demonstrating the variations in the parameters observed in corn plants and allowing us to observe the relationships between the variables and the hydric and inoculation treatments. Most variables correlated significantly (*p* < 0.05) with the first axis of PCA. In contrast, variables such as A/E, NPQ, qN, Fo, Ca, % Col. and Clt/Carot were negatively correlated with this axis (Fig. 6).

**Figure 7:**
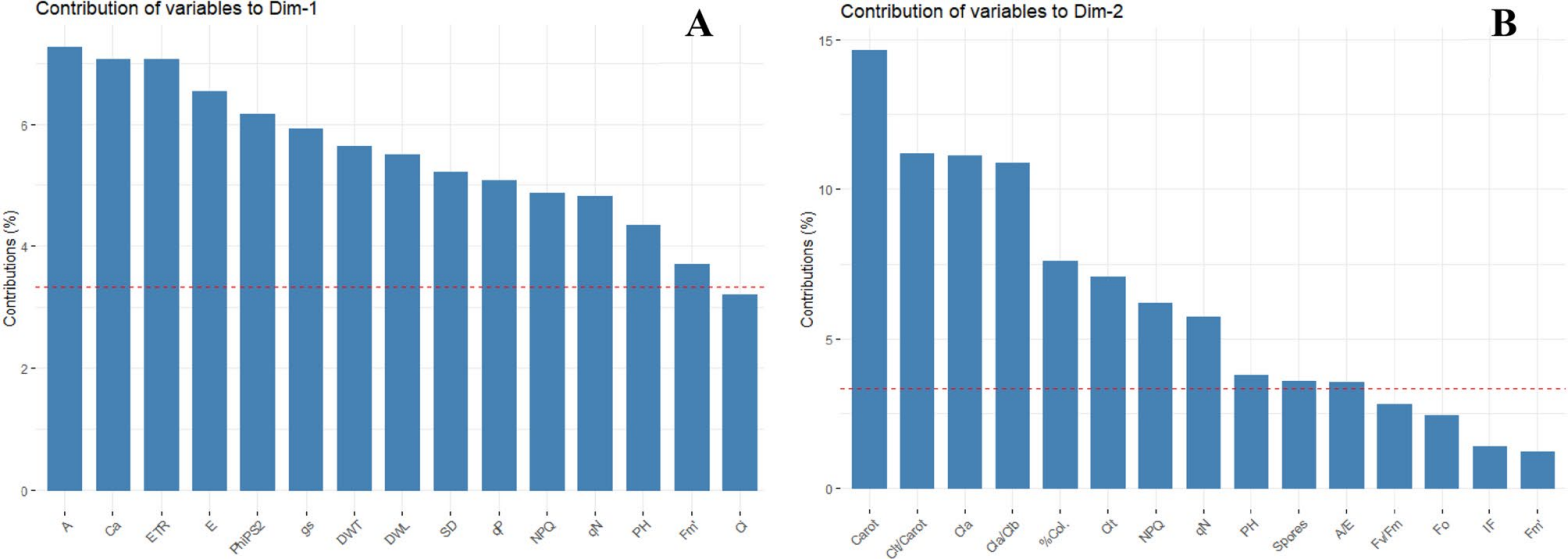
Contributions of variables in corn plants *(Zea mays)* submitted to different water treatments and inoculated with AMF after reirrigation. First dimension (**A**) and second dimension (**B**). A, Photosynthetic rate; Ca, external concentrations of CO_2_; ETR, Electron transport; E, transpiration; PhiPS2, effective photochemical efficiency of photosystem II; gs, stomatal conductance; DWT, total dry weight; DWL, dry leaf weight; SD, stem diameter; qP = , PH, Plant height; qP, photosynthetic quenching; Ci, internal concentrations of CO_2_; Carot, carotenoid; Clt/Carot, total chlorophyll/carotenoid; Cl*a*, Chlorophyll *a*; Cl*a*/Cl*b*, Chlorophyll *a*/ Chlorophyll *b*; %Col., mycorrhizal colonization; Clt, total chlorophyll; NPQ, nonphotochemical extinction coefficient; qN, nonphotochemical quenching; Spores, spore density; A/E, water use efficiency; Fv/Fm, maximal quantum yield of PSII; Fo, minimum fluorescence; IF, phaeophytinization index; Fm’, maximal fluorescence level in the light-adapted state.

Regarding the individual contribution of the variables in PC1, the following trend was observed: A > Ca > ETR > E > PhiPS2 > gs > DWT > DWL > SD > qP > NPQ > qN > PH > Fm' > Ci. Different from the trend observed in PC2: Carot > Clt/Carot > Cla > Cla/Clb > % Col. > Clt > NPQ > qN > PH > Spores > A/E > Fv/FM > Fo > IF > Fm' (Fig. 7).

**Figure 8:**
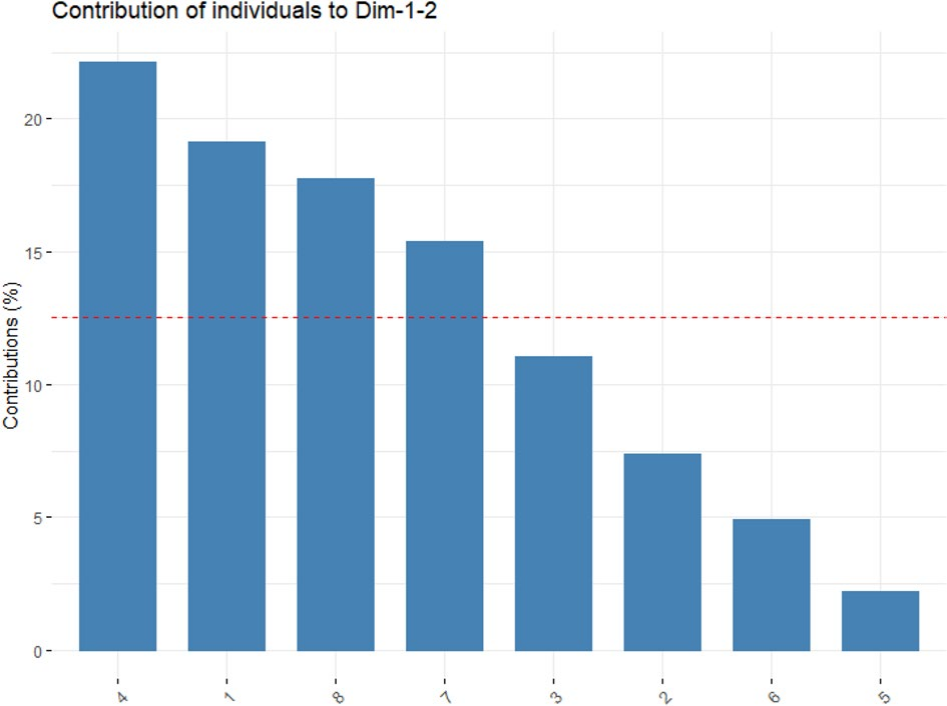
Graphical illustration of individual contribution of the treatments to PCs. (1) Control—NI; (2) Control—HD; (3) *Claroideoglomus etunicatum*—NI; (4) *Claroideoglomus etunicatum*—HD; (5) *Rhizophagus clarus*—NI; (6) *Rhizophagus clarus*—HD; (7) *Rhizophagus intraradices*—NI; (8) *Rhizophagus intraradices*—HD. *HD* Hydric deficit and *NI* normaly irrigated.

Meanwhile, for the individual contribution of treatments to the total variation (PC1 and PC2), the following trend was observed: *Claroideoglomus etunicatum*—HD > Control—NI > *Rhizophagus intraradices*—HD > *Rhizophagus intraradices*—NI > *Claroideoglomus etunicatum*—NI > Control—HD > *Rhizophagus clarus*—HD > *Rhizophagus clarus*—NI (Fig. 8).

## Discussion

Drought affects plant growth, reducing leaf water potential, cell division^13^, and photosynthetic plant capacity. In the present study, the water potential (Ψw) of plants differed between the water and inoculation treatments (Fig. 1A). Plants under HD showed no difference between them. For irrigation, the treatment inoculated with *R. intraradices* (ROOTELLA BR) obtained a higher Ψw, differing from the other. The decrease in water potential results in low cellular turgor and a reduction in cell division^13^, culminating in low stomatal conductance and directly affecting the photosynthetic rate, which results in slower plant growth^41^.

It was observed that there was a higher number of AMF spores in plants inoculated mainly with *C. etunicatum* in the HD treatment (Fig. 1B); however, it did not differ from those inoculated with *R. clarus*. In the irrigated water treatment, the highest density of spores was observed for plants inoculated with *R. intraradices* (ROOTELLA BR). Mycorrhizal colonization (Fig. 1C) was higher mainly in plants inoculated with the AMF *C. etunicatum* but did not differ from those inoculated with *R. intraradices* (ROOTELLA BR) and *R. clarus*. The percentage of colonization in plants was not so high, and this limitation of root colonization when the plant is under hydric deficit can occur for several reasons, including a decrease in germination and spore growth, decrease in the number of AMF, inhibition in growth and dissemination of hyphae in soil^42^, decline in carbohydrate supply by host plants^43^, where the corn exudes carboxylates poorly^44^ and the high level of phosphorus in the soil having a negative influence on the mycorrhizal response^45^, providing reduction in symbiosis^46^. In addition, the type of plant and fungus, cultivation conditions and exposure time interfere with the colonization potential^47^.

The photosynthesis of HD-treated plants (Fig. 2A) was able to increase its rate after the stress period and may be related to a greater stomatal conductance, especially in plants inoculated with *R. clarus* and *R. intraradices* (ROOTELLA BR) (Fig. 2B). The transpiration rate showed differences only for the hydric treatments (Fig. 2C). Low photosynthetic yields may be related to lower stomatal opening in plants submitted to water deficit, reducing CO_2_ input, but plants associated with AMF maintain or increase stomatal conductance when compared to noninoculated plants. This characteristic can be observed in the present work in plants of corn inoculated with *R. clarus* and *R. intraradices*, and the same is mentioned by^47^ working with soybean cultivars subjected to water deficit and inoculated with *R. clarus*.

The disorientation of photosynthesis is associated with low electron transport through PSII (ETR) and/or structural injury of PSII and reaction centers^48^. However, soon after reirrigation, the ETR (Fig. 3A) of irrigated plants showed no difference. For plants that were previously submitted to HD, the electron transport rate showed good behavior. Plants inoculated with *R. clarus* and *R. intraradices* (ROOTELLA BR), which previously had the lowest values, were able to increase their rate, sticking out those inoculated with *C. etunicatum.* This same behavior can be observed for PhiPS2 (Fig. 3C).

The maximum quantum yield of PSII (Fv/Fm) showed differences for hydric treatments (Fig. 3B), in which HD plants were smaller than irrigated plants. However, all of them presented an average value greater than 0.75 quantum electrons^−1^, indicating that the evaluated plants did not suffer photoinhibitory damage after reirrigation. This value is indicated as a reference to indicate stress^49^.

According to some authors, when plants have values lower than 0.75 quantum electrons^−1^, they indicate a situation of stress and reduction of photosynthetic potential. When values range from 0.75 to 0.85 quantum electrons^−1^, it may suggest that the photosynthetic device did not suffer significant damage^50–52^ found that poplars (*Populus* spp.) inoculated with the AMF *Rhizophagus irregularis* showed drought tolerance, and the fungus collaborated so that there was no decline in the levels of Fv/Fm and qP.

Nonphotochemical extinction (NPQ), which reflects the heat dissipation capacity when there is excess light energy, can reduce the transfer of energy to reaction centers without any effect on qP (degree of closing of the reaction center)^53^. In the present study, NPQ differed separately for hydric and inoculation treatments (Fig. 3D), in which plants under HD presented higher heat dissipation capacity. Concerning inoculation treatments, *R. intraradices* (ROOTELLA BR) presented a lower mean NPQ value. A high level of NPQ may result from a low electron transport rate (ETR), which impedes the formation of reactive oxygen species (ROS)^54^. ROS can directly contribute to damage to PSII or inhibit the repair of reaction centers^55^ and are formed through excessively absorbed excitation energy, causing impairment of photosynthetic function and leading to an accumulation of ROS, resulting in oxidative stress^56^. The increase in NPQ in plants under hydric stress indicates a possible decrease in the photosynthetic process and the fixation of CO_2_. This damage can decrease the use of electron transport products, culminating in greater thermal dissipation.

Chlorophyll *a* remained constant, in which only plants inoculated with *R. intraradices* (ROOTELLA BR) under HD presented a lower rate (Fig. 4A). Mathur et al.^57^ observed in a study conducted with wheat (*Triticum aestivum*) that water stress reduced the concentration of total chlorophylls in plants under HD without inoculation, but mycorrhized plants were able to maintain their pigment concentration high because these plants were able to obtain more water through hyphae.

Plants when they are in a long period of stress end up losing leaf area to reduce transpiration to protect plants from possible oxidative damage caused by a smaller surface area of light; however, such changes may mean lower biomass production^41^. Height (Fig. 5A) and diameter (Fig. 5B) showed differences only for water treatments, in which HD plants had the lowest rates when compared to irrigated plants.

Plants under hydric stress have lower height and diameter and, consequently, lower dry weight of leaves and roots, demonstrating that HD affects biomass production. It is noted that damage to the water status of the plant directly affects its growth and development. The dry weight of leaves (Fig. 5C) and roots (Fig. 5D) showed that HD plants presented lower weight than irrigated plants, culminating in a lower total dry weight in plants under stress (Fig. 5E).

Water stress can lead to disruption of lipids and membrane proteins and, eventually, to changes in enzymatic activities^58^. Membrane permeability is generally evaluated as electrolyte leakage measured through free and total electrical conductivity and is an essential indicator of plant cell membrane health in stressful conditions^59^. In plants grown under conditions of hydric stress, cell membrane stability or membrane damage are the most important parameters of plant cell response and tolerance to abiotic stress species and can be used as indicators of plant cell membrane integrity^60^. This parameter can be evaluated by leaking electrolytes in plants subjected to water stress. In the present study, free electrical conductivity showed no difference between the hydric and inoculation treatments, which may indicate that there was no damage to the cell membrane.

In general, inoculation with AMF provided benefits to corn plants, helping to recover from the stress caused, maintaining or increasing their development in drought conditions. Understanding the development and physiological behavior of maize plants associated with AMF, among these potential inoculants, provides information for future field research and strategies to mitigate the effects of drought and consequently reduce grain yields.

## Conclusions

The corn inoculated with AMF showed benefits in development and recovery because the fungi helped to tolerate the hydric restriction period by maintaining the functioning of the photosynthetic apparatus of the plants.

## Supplementary Information

Supplementary Information.
