# Supplementary material for: Arbuscular mycorrhizal fungi associated with maize plants during hydric deficit
Source: Sci Rep. 2023 Jan 27;13:1519. doi: 10.1038/s41598-023-28744-4 (PMC9883248; doi:10.1038/s41598-023-28744-4)
Supplement: Supplementary file 1 — Supplementary Information. [file 41598_2023_28744_MOESM1_ESM.docx]

**Arbuscular mycorrhizal fungi associated with maize plants during hydric deficit**

Letícia Rezende Santana^1^; Lais Noamy da Silva^1^; Germanna Gouveia Tavares^1^; Priscila Ferreira Batista^1^; Juliana Silva Rodrigues Cabral^2*^; Edson Luiz Souchie^1^

^1^Instituto Federal Goiano, Campus Rio Verde, Rodovia Sul Goiana km 01, Cx. P. 66.CEP 75901-970, Rio Verde - GO.

^2^Faculdade de Agronomia, Universidade de Rio Verde, Fazenda Fontes do Saber -Campus Universitário - Rio Verde Goiás, Cx Postal: 104 - CEP 75901-970.

*email: jsrcabral@gmail.com

**Supplementary Material**

**Table 1S.** Soil analysis (0-20 cm) before the installation of the experiment, in Rio Verde, GO.

| **Ca** | **Mg** | **Ca+Mg** | **Al** | **H+Al** | **K** | **K** | **S** | **P (mel)** | **pH** | **O.M.** |
| --- | --- | --- | --- | --- | --- | --- | --- | --- | --- | --- |
| **----------------------cmol_c_ dm^-3^---------------------** | | | | | | **------mg dm^-3^------** | | | **CaCl_2_** | **g dm­­^-3^** |
| 3.64 | 1.49 | 5.13 | 0.00 | 6.15 | 0.23 | 88.00 | 5.73 | 2.50 | 4.78 | 49.00 |
| **Na** | **Fe** | **Mn** | **Cu** | **Zn** | **B** | **CEC** | **SB** | **V%** | **m%** | **Clay** |
| **------------------mg dm^-3^------------------** | | | | | | **cmol_c_ dm^-3^** | |  |  | **(g dm^-3^)** |
| 1.00 | 5.68 | 13.68 | 1.02 | 1.51 | 0.18 | 11.50 | 5.36 | 46.56 | 0.00 | 440.00 |

Ca= calcium; Mg= magnesium; Al= aluminium; H + Al – potential acidity; K= potassium; S= súlfur; P (mel)= phosphorus (mehlich); O.M= organic matter; Na= sodium; Fe= iron; Mn= manganese; Cu= Copper; Zn= zinc; B= boron; CEC= cation exchange capacity; BS= base sum; V%= base saturation percentage; m%= aluminum saturation percentage; mg= milligram; dm= decimeter; cmol_c_= centimole of charge
